# 1653. Transcriptional Variation of Adhesion and Biofilm Genes in CTX-M-15-Expressing *Klebsiella pneumoniae*

**DOI:** 10.1093/ofid/ofac492.119

**Published:** 2022-12-15

**Authors:** Alyssa K W Maclean, Nancy D Hanson

**Affiliations:** Creighton University, Omaha, NE; Creighton University, Omaha, NE

## Abstract

**Background:**

*Klebsiella pneumoniae* can produce a number of β-lactamases, including the cefotaximase, CTX-M-15. While classically associated with ST131 *Escherichia coli*, CTX-M-15 is associated with increased virulence in *K. pneumoniae*. Clinical isolates of *K. pneumoniae* have been shown to produce high levels of CTX-M-15 RNA. But, the overall transcriptomic changes due to CTX-M-15 expression are unknown. The purpose of this study was to determine if acquisition of CTX-M-15 altered the global transcriptome of *K. pneumoniae* in addition to decreasing β-lactam susceptibility.

**Methods:**

The structural gene and native promoter of CTX-M-15 were cloned into a low copy number vector and transformed into Kp 23, a clinical isolate from the 1970s with no known acquired β-lactam resistance genes. CTX-M-15 production was confirmed by qRT PCR and western blot. MICs to cefotaxime, ceftazidime, cefepime, ceftolozane/tazobactam, ceftazidime/avibactam, meropenem, meropenem-vaborbactam, and imipenem-relebactam were determined by E-test according to CLSI guidelines. RNA-Seq analysis and bioinformatics were performed by MiGS.

**Results:**

CTX-M-15 carriage led to non-susceptible MICs to cefotaxime, ceftazidime, and cefepime. CTX-M-15 production was associated with statistically significant upregulation of 204 genes and downregulation of 231 genes. Among these, the biofilm genes *mrkA* and two copies of *mrkB* were downregulated by 7.5, 4 and 3.5-fold, respectively. Additionally, *ecpA, ecpB, ecpC, and ecpR –* components of the *E. coli* Common Pilus (ECP) – were upregulated by 3, 2, 2, and 3-fold, respectively.

**Conclusion:**

High-level expression of CTX-15 β-lactamase has a significant impact on the transcriptome of *K. pneumoniae*. These changes include a down regulation of genes associated with biofilm formation with a concomitant increase in the expression of genes associated with adherence. These data indicate that overexpression of CTX-M-15 and perhaps other β-lactamases may help promote the pathogenicity of *K. pneumoniae* or influence its ability to colonize the tissue it infects. This may be important during urinary tract infections when an increase in adhesion by CTX-M-15 producing isolates may be required over biofilm formation to establish an active infection.

**Disclosures:**

**Nancy D. Hanson, PhD**, Merck: Grant/Research Support.

